# Characterization of Sn_4_P_3_–Carbon Composite Films for Lithium-Ion Battery Anode Fabricated by Aerosol Deposition

**DOI:** 10.3390/nano9071032

**Published:** 2019-07-19

**Authors:** Toki Moritaka, Yuh Yamashita, Tomohiro Tojo, Ryoji Inada, Yoji Sakurai

**Affiliations:** 1 Department of Electrical and Electronic Information Engineering, Toyohashi University of Technology, 1-1 Tempaku-cho, Toyohashi, Aichi 4418580, Japan; 2 Department of Electrical and Electronic Engineering, Shizuoka Institute of Science and Technology, 2200-2 Toyosawa, Fukuroi, Shizuoka 437-8555, Japan

**Keywords:** tin phosphide, carbon, composite film, aerosol deposition, lithium-ion battery, anode

## Abstract

We fabricated tin phosphide–carbon (Sn_4_P_3_/C) composite film by aerosol deposition (AD) and investigated its electrochemical performance for a lithium-ion battery anode. Sn_4_P_3_/C composite powders prepared by a ball milling was used as raw material and deposited onto a stainless steel substrate to form the composite film via impact consolidation. The Sn_4_P_3_/C composite film fabricated by AD showed much better electrochemical performance than the Sn_4_P_3_ film without complexing carbon. Although both films showed initial discharge (Li^+^ extraction) capacities of approximately 1000 mAh g^−1^, Sn_4_P_3_/C films retained higher reversible capacity above 700 mAh g^−1^ after 100 cycles of charge and discharge processes while the capacity of Sn_4_P_3_ film rapidly degraded with cycling. In addition, by controlling the potential window in galvanostatic testing, Sn_4_P_3_/C composite film retained the reversible capacity of 380 mAh g^−1^ even after 400 cycles. The complexed carbon works not only as a buffer to suppress the collapse of electrodes by large volume change of Sn_4_P_3_ in charge and discharge reactions but also as an electronic conduction path among the atomized active material particles in the film.

## 1. Introduction

Li-ion batteries (LiBs) are widely used as a power source for portable electronic devices, and recently have attracted much attention as a large-scale power source for electric vehicles and plugin hybrid electric vehicles. In order to achieve advanced LiBs with higher energy density, development of anode materials with higher capacity is indispensable. Graphite with a theoretical capacity of 372 mAh g^−1^ is commonly used as an anode for LiBs, while lithium alloys such as Li–Si and Li–Sn with a higher theoretical capacity (Li_4.4_Si: 4200 mAh g^−1^, Li_4.4_Sn: 990 mAh g^−1^) have been extensively studied [1,2,3]. However, they result in poor cycling stability due to a large volume change during charge and discharge reactions. In order to improve the cycling stability, various composite materials including metal oxides, multiphase alloys and intermetallic compounds have been studied as alternatives to graphite anode for LiBs [3,4,5,6,7]. These materials show much higher capacities than graphite and improved cycling performance compared to lithium alloy materials. The enhancement of cycling stability in these Li-alloy-based materials attributed to an inactive matrix [4]. Li-alloy-based materials form an inactive matrix during cycling and this matrix is expected to suppress the volume change of the alloying reaction, and keeps the electrode particles mechanically connected together resulting in a reversible alloying reaction.

Tin phosphide Sn_4_P_3_ (theoretical gravimetric capacity = 1255 mAh g^−1^) is known as one of the high capacity alloy-based anode materials for LiBs [8,9,10,11]. Sn_4_P_3_ has a layered structure (space group: R-3m) suitable for lithium insertion and high intrinsic electronic conductivity at room temperature. In addition, Sn_4_P_3_ forms Sn and Li_3_P in the lithium insertion reaction. Li_3_P has high ionic conductivity [12] and would act as a matrix for suppressing the volume change during the alloying reaction. According to these features, application of a Sn_4_P_3_ anode for high-capacity sulfide-based solid-state batteries has been also demonstrated [13].

As reported in the literature [8,9], Sn_4_P_3_ shows initial reversible capacity as high as 900 mAh g^−1^ and by controlling the electrical potential window in galvanostatic charge and discharge testing, it maintains a reversible capacity above 400 mAh g^−1^ after 50 cycles. Reducing the size and morphology of Sn_4_P_3_ particles [14,15] and doping of a small amount of Fe [16] and Mn [17] into Sn_4_P_3_ are also effective for further improvement of the cycling stability. Moreover, complexing the carbon materials with nano-structured Sn_4_P_3_ particles significantly enhances both the rate performance and cycling stability [18,19,20,21,22,23,24,25]. The complexed carbon behaves as the buffer for the volume change of active material particles during charge and discharge reaction and maintains the electric conduction between the particles.

In general, the electrodes used in actual batteries are fabricated by coating a slurry composed of electrode active materials on metallic foils and contain conducting carbon additives and binders. For the case of carbon complexed alloy-based anodes, the weight fraction of active materials in an electrode becomes small (less than ~70%) due to significant amounts of conducting additives and binders. Consequently, gravimetric specific capacity calculated by the total mass of the electrode (including carbon additives and binders) is reduced significantly. To address this issue, we are focusing on the aerosol deposition (AD) method [26,27,28] as an electrode fabrication process, which uses impact consolidation for ceramic particles at room temperature. This method is known as a fabrication process of various functional ceramic films at room temperature. By controlling the size and morphology of the base powder material, the film fabricated by AD has a dense structure made of nanocrystalline particles, and the structural and physical properties are similar to the base powder material. Moreover, adhesion strength between the film formed by AD and the substrate is high without adding binders [26,27]. To date, several works for the application of AD to rechargeable battery materials have been reported. The electrochemical performance for film-shaped electrodes of Si alloy or composite [29,30], tin-phosphide with different compositions [31], transition metal oxides [32,33,34,35,36,37,38,39,40] formed on a metal and a ceramic-based solid electrolyte substrate have been studied to verify the feasibility of AD. Moreover, as-deposited solid electrolyte films show a moderate Li^+^ conductivity of 10^−7^–10^−5^ S cm^−1^ at room temperature [41,42,43,44,45].

In this work, we fabricated Sn_4_P_3_–carbon (Sn_4_P_3_/C) composite films on a stainless steel substrate by AD and the electrochemical performance of the LiB anode was evaluated. Sn_4_P_3_/C composite powder was prepared by ball milling and used as a raw material to form the composite film via impact consolidation. The influence of complexed carbon on the cycling stability of both the microstructure and reversible capacity was examined.

## 2. Materials and Methods

### 2.1. Fabrication and Characterization of Sn_4_P_3_/C Composite Powders

Sn_4_P_3_ powder was prepared using a simple mechanochemical synthesis with a planetary ball-milling [8,9,13]. Sn (99%, Kojundo Chemical Laboratory, Saitama, Japan) and red P (99.9%, Kojundo Chemical Laboratory, Saitama, Japan) powders were used as starting materials. Stoichiometric amounts of the starting materials (10 g) were put into a ZrO_2_ vessel (45 mL) with ZrO_2_ balls that were 10 mm in diameter (100 g) and reacted in a planetary ball milling apparatus (Nagao System, Planet M2-3F, Kawasaki, Japan) with a fixed rotation speed of 350 rpm for 8 h under an Argon atmosphere.

It is known that controlling the particle size of raw powder is important for film fabrication by AD [14,15,16,28,30,32], and we could not form Sn_4_P_3_ film by AD with as-synthesized powder. In order to prepare Sn_4_P_3_ powders suitable for AD, as-synthesized Sn_4_P_3_ powder (~10 g) was put into a ZrO_2_ vessel with ethanol (30 mL) and ZrO_2_ balls with diameters of 1 mm (30 g) and 2 mm (100 g) and then pulverized by a planetary ball-milling at 350 rpm and 24 h. After the pulverization, the obtained Sn_4_P_3_ powder and acetylene black (AB) were mixed with a weight ratio of Sn_4_P_3_:AB = 8:2. The mixture (~5 g) was put into a ZrO_2_ vessel again with ZrO_2_ balls with diameters of 5 mm (50 g) and 10 mm (100 g), and Sn_4_P_3_/C composite powder was prepared by a planetary ball-milling at 350 rpm for 24 h.

The crystal phase of as-synthesized Sn_4_P_3_, ball-milled Sn_4_P_3_ and Sn_4_P_3_/C powder was evaluated by X-ray Diffractometer (XRD; Rigaku, MultiFlex, Tokyo, Japan) using Cu Kα radiation (λ = 0.15418 nm), with a measurement range 2θ of 5–90° and a step interval of 0.002°. Field emission scanning electron microscopy (FE-SEM; Hitachi High-Technologies, SU8000 Type II, Tokyo, Japan) was used to observe the size and morphology for all powder samples. Energy dispersive X-ray (EDX) analysis was also performed using FE-SEM, to observe Sn_4_P_3_/C particles and the corresponding distribution of Sn, P and C elements.

### 2.2. Fabrication and Characterization of Sn_4_P_3_/C Composite Films by AD

As shown in the literature [34,40,42], an AD apparatus consists of a carrier gas supplying system, an aerosol chamber, a deposition chamber equipped with a motored *X-Y-Z* stage and a nozzle with a thin rectangular-shaped orifice with the cross-sectional size of 10 mm × 0.5 mm. Sn_4_P_3_/C powder was used as a raw material for fabricating Sn_4_P_3_/C composite film by AD. A carrier nitrogen (N_2_) gas flows out from the gas supply system to the aerosol chamber. In the aerosol chamber, the powder is dispersed into the carrier gas to make an aerosol. Using a pressure difference between the evacuated deposition chamber and the carrier gas system, the aerosol flows into the deposition chamber through a nozzle and is sprayed onto an SUS316L stainless steel substrate. The deposition area was masked into a circular shape 8 mm in diameter. The deposition chamber was evacuated to a low vacuum state at approximately 20 Pa and deposition was carried out for 20–30 min. During the deposition process, the stage was moved uni-axially with a back-and-forth motion length of 50 mm and a speed of 10 mm s^−1^. Based on the results in our previous works [34,38,40,42,44], the distance between the substrate and nozzle tip was set to 10 mm and the mass flow of the N_2_ carrier was fixed at 20 L min^−1^.

The crystal phase of the Sn_4_P_3_/C composite film was evaluated by XRD using Cu Kα radiation (λ = 0.15418 nm), with a measurement range 2θ of 5–90° and a step interval of 0.002°. Microstructure observation of composite films was carried out by using FE-SEM, and EDX analysis was also performed to observe the distribution of Sn, P and C elements in the composite film.

The electrochemical properties of as-deposited Sn_4_P_3_/C films were evaluated by using a two-electrode set up. A Sn_4_P_3_/C film on an SUS316L substrate was used as a working electrode, where as a single lithium foil served as both a counter and a reference electrode. The electrolyte solution was 1 mol L^−1^ LiPF_6_ in a mixture or ethylene carbonate (EC) and dimethyl carbonate (DMC) with a volume ratio of 1:1 (Kishida Chemical Co., Ltd., Osaka, Japan). Together with a separator (Celgard, Celgard 3501, Tokyo, Japan), these components were assembled in a CR2032 coin-type cell. The cell assembly was carried out in a dry Argon-filled glove box. The cells were charged and discharged over the cell voltage ranges of 0 to 0.75, 1, 1.25 and 2.5 V at a fixed current density (per total mass of composite film) of 50 mA g^−1^ and 25 °C using the Battery Test System (TOSCAT-3100, TOYO-SYSTEM, Iwaki, Japan). After the cycling test, the cells were disassembled in a dry Argon-filled grove box and the microstructure of the Sn_4_P_3_/C composite films was observed using FE-SEM. Before the observations, cycled Sn_4_P_3_/C films were cleaned with DMC to eliminate residual Li salt therein.

## 3. Results and Discussion

### 3.1. Crystal Phase and Microstructure of Sn_4_P_3_/C Composite Powder and Film

**Figure 1:**
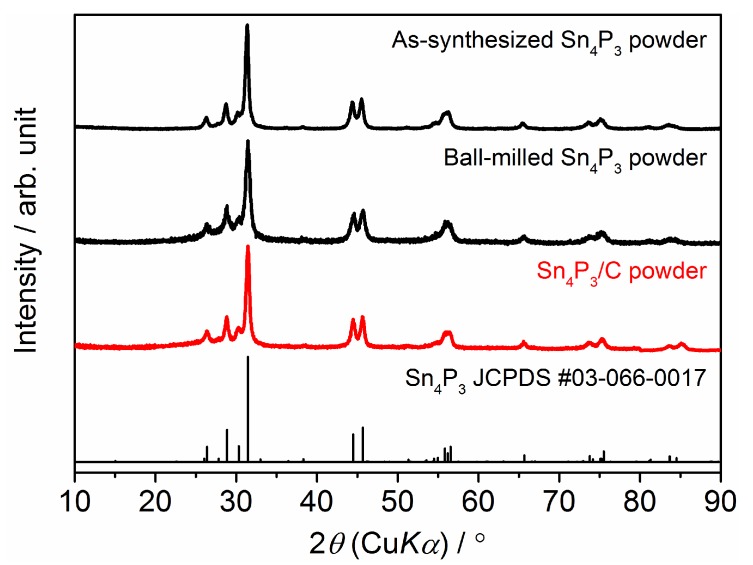
Comparison of X-ray diffraction (XRD) patterns for as-synthesized Sn_4_P_3_ powder, ball-milled Sn_4_P_3_ powder and Sn_4_P_3_/C composite powder.

**Figure 2:**
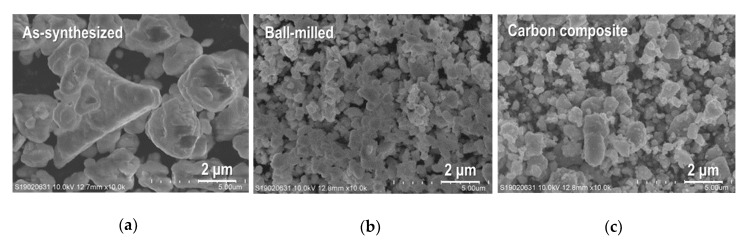
Scanning electron microscopy (SEM) images for (**a**) as-synthesized Sn_4_P_3_ powder, (**b**) ball-milled Sn_4_P_3_ powder and (**c**) Sn_4_P_3_/C composite powder.

The XRD data for as-synthesized Sn_4_P_3_, ball-milled Sn_4_P_3_ and Sn_4_P_3_/C composite powders are summarized in Figure 1. The pattern for Sn_4_P_3_ (JCPDS No. 03-066-0017) is also shown as the reference. It is confirmed that peak patterns for all sample powders agree well with the reference, suggesting that any structural changes did not occur in the ball-milling and carbon-complexing process. No peaks from carbon were detected in the Sn_4_P_3_/C powder because complexed carbon with Sn_4_P_3_ has amorphous structure. Figure 2 shows scanning electron microscopy (SEM) images for as-synthesized Sn_4_P_3_, ball-milled Sn_4_P_3_ and Sn_4_P_3_/C composite powders. As-synthesized powder consists of agglomerated particles with a size of 1–5 µm (Figure 2a). After ball-milling, the particle size reduces to 0.5–1.5 µm (Figure 2b). On the other hand, after complexing carbon (acetylene black (AB)) with Sn_4_P_3_ by ball-milling, the particle size is not changed remarkably (Figure 2c).

**Figure 3:**
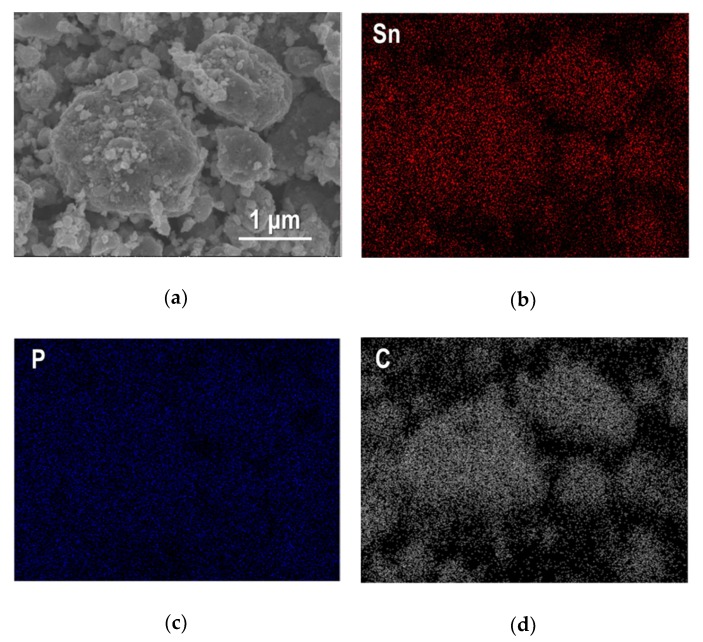
(**a**) SEM image of Sn_4_P_3_/C composite powder and corresponding element mapping for Sn, P and C are shown in (**b**), (**c**) and (**d**).

A higher magnified SEM image of Sn_4_P_3_/C particles and elementary distributions for Sn, P and C in an observation area are shown in Figure 3. As shown in Figure 3a, the sample powder looks like agglomerated particles with a particle size of about 0.5–2 μm. Sn and P show similar distribution in an observed area and were detected on agglomerated particles, and interestingly, C is also distributed along the particle shape. This suggests that carbon (AB) particles are complexed successfully with Sn_4_P_3_ particles by a simple ball-milling process.

**Figure 4:**
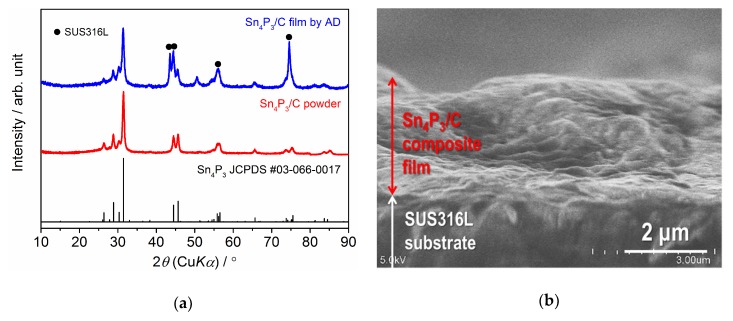
(**a**) XRD patterns for the Sn_4_P_3_/C composite powder and film formed on an SUS316L substrate and (**b**) cross-sectional SEM image for the Sn_4_P_3_/C composite film formed on an SUS316L substrate.

**Figure 5:**
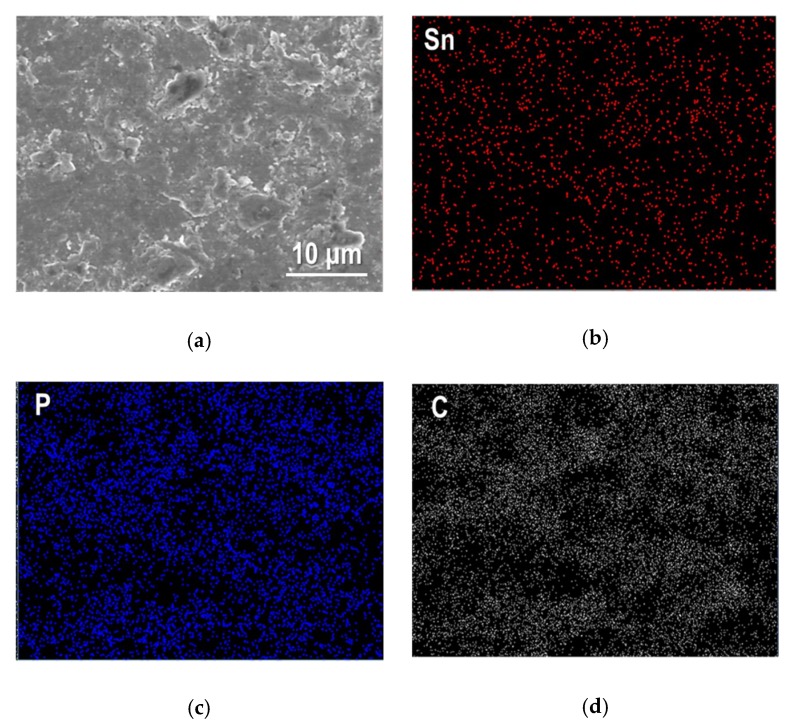
(**a**) SEM image of the broader surface of the Sn_4_P_3_/C composite film. Corresponding element mapping for Sn, P and C are shown in (**b**), (**c**) and (**d**).

Figure 4 shows the XRD pattern and cross-sectional SEM image of the Sn_4_P_3_/C composite film formed on an SUS316L substrate by AD. Note that the peak intensity for the composite film is one order lower than the raw powder. Together with the peaks from the substrate, the peaks from the Sn_4_P_3_ phase are clearly confirmed but become broader compared to raw powder. No other secondary phases are formed during film fabrication by AD. The thickness of the composite film is confirmed to be 2.5–3 µm. An SEM image and elementary distributions for Sn, P and C for the broader surface of the Sn_4_P_3_/C composite film are shown in Figure 5. It can be seen that the film is composed of deformed or fractured particles. Moreover, Sn, P and C are distributed uniformly in an observed area, suggesting that carbon is included successfully in the film.

### 3.2. Electrochemical Performance of Sn_4_P_3_ Film Electrodes

**Figure 6:**
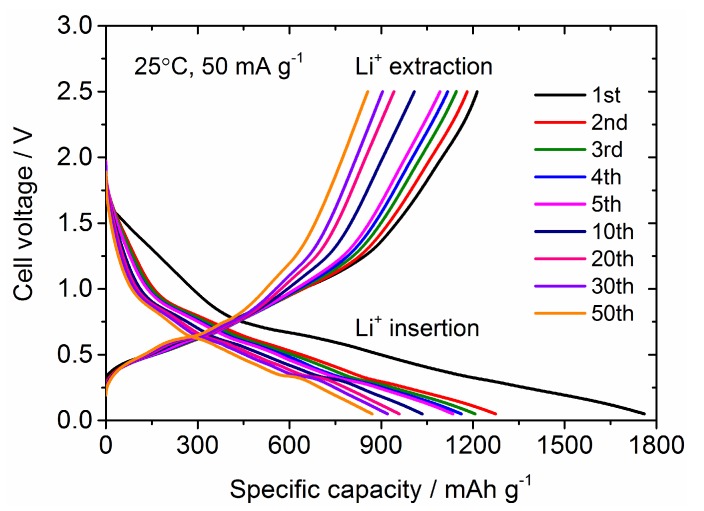
Galvanostatic charge and discharge curves at different cycle numbers for the Sn_4_P_3_/C composite film electrode at 25 °C and 50 mA g^−1^. Cell voltage window for galvanostatic cycling is 0–2.5 V.

Next, we discuss the electrochemical performance for Sn_4_P_3_/C composite film as a LiB anode. Figure 6 shows the galvanostatic charge (Li^+^ insertion) and discharge (Li^+^ extraction) curves at different cycle numbers for the Sn_4_P_3_/C film electrode in a coin-type cell. The cell voltage window is from 0 to 2.5 V. At the first cycle, charge capacity reaches 1750 mAh g^−1^, which is much higher than the theoretical capacity (1255 mAh g^−1^) of Sn_4_P_3_, while discharge capacity is confirmed to be 1200 mAh g^−1^. Coulombic efficiency at the first cycle is 69%, but irreversibility in charge and discharge reaction is greatly reduced after the second cycle and the Coulombic efficiency retains 96–98%. This suggests that the charge capacity in the first cycle includes the contribution of side reactions such as the decomposition of an organic liquid electrolyte at a lower cell voltage to form Li^+^ conducting solid-electrolyte interphase (SEI) on the electrode surface. The reversible capacity decreases monotonically with cycling but retains a high capacity of 800 mAh g^−1^ at the 50th cycle. The averaged operation potential is approximately 0.7 V and step-like profiles are confirmed in both charge and discharge processes, which is consistent with the results reported in the literature [8,9,10,11].

**Figure 7:**
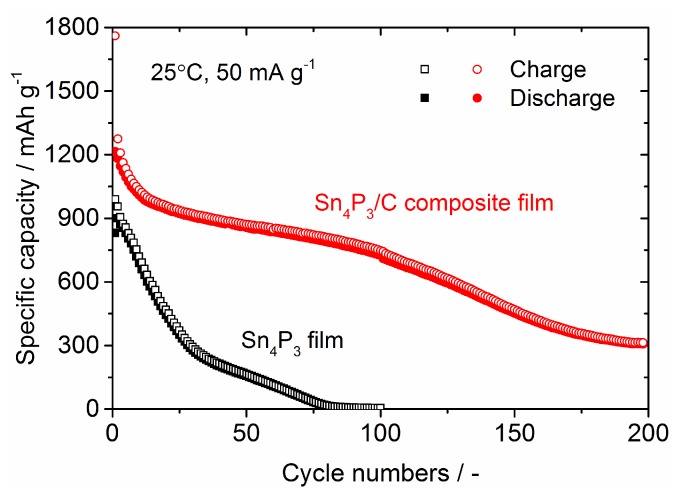
Comparison of cycling stability of charge and discharge capacities for the Sn_4_P_3_/C composite film and the Sn_4_P_3_ film electrodes. Cell voltage window for galvanostatic cycling is 0–2.5 V.

Cycling stability for the Sn_4_P_3_/C composite film electrode is shown in Figure 7, together with the data for the Sn_4_P_3_ film electrode without complexing carbon. The Sn_4_P_3_ film was fabricated by AD with ball-milled Sn_4_P_3_ powder (Figure 2b) on an SUS316L substrate, with a thickness of approximately 2 µm (Figure S1). As can be seen, the capacity fading in the Sn_4_P_3_ film with cycling is relatively fast and reduces to less than 10 mAh g^−1^ at the 80th cycle. We checked the Sn_4_P_3_ film taken from a disassembled coin-type cell after testing and confirmed that the majority of the film was collapsed and peeled off from the SUS316L substrate. Therefore, the rapid degradation of Sn_4_P_3_ films with cycling is caused by the mechanical damage due to the large volume change of the Sn_4_P_3_ film in charge and discharge reactions. On the other hand, the Sn_4_P_3_/C composite film shows much better cycling stability than the Sn_4_P_3_ film and retains a reversible capacity above 700 mAh g^−1^ even at the 100th cycle, indicating that the complexed carbon is effective at enhancing the cycling performance of Sn_4_P_3_. However, the capacity fading is accelerated by further cycling above 100 cycles and the reversible capacity is reduced to 300 mAh g^−1^ at the 200th cycle. It is worth noting that we also fabricated a Sn_4_P_3_/C composite film with a lower carbon content (weight ratio Sn_4_P_3_:C = 9:1) and evaluated the electrochemical performance, but the degradation with cycling becomes more significant and the reversible capacity at 100 cycles was reduced to 500 mAh g^−1^ (Figure S2).

**Figure 8:**
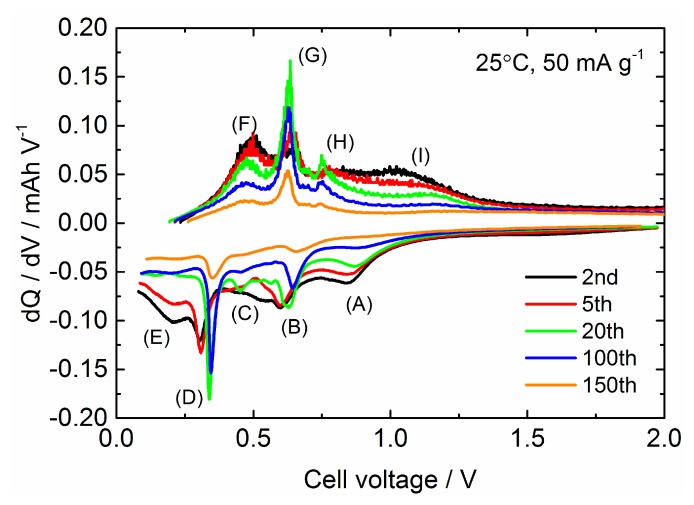
Differential capacity d*Q*/d*V* (*Q*: capacity, *V*: cell voltage) curves for the Sn_4_P_3_/C composite film calculated from the data for galvanostatic cycling test.

As shown in Figure 7, the degradation mode of the Sn_4_P_3_/C composite film in galvanostatic cycling seems to be classified into three processes: (1) Rapid degradation below 15 cycles, (2) gradual degradation from 15 to 100 cycles, (3) accelerated degradation after 100 cycles. In order to further examine the degradation process, differential capacities d*Q*/d*V* (*Q*: capacity (mAh), *V*: cell voltage (V) are calculated and plotted against a cell voltage in Figure 8. Based on the examination of the electrochemical reaction mechanism for Sn_4_P_3_ anode characterized by ex-situ XRD and X-ray absorption spectroscopy (XAS) analysis in the literature [8], the reactions occurring in the charge process are considered as follows: (A) Li^+^ insertion into the layered structure of Sn_4_P_3_ (at ~0.85 V), (B) Formation of LiP and partial transformation from LiP to Li_3_P (at ~0.65 V), (C) and (D) Alloying of Sn with Li to form Li*_x_*Sn alloy (at ~0.5 V and ~0.3 V) and (E) Formation of Li_3_P and Li*_x_*Sn (at <0.25 V). On the other hand, reactions in the discharge process are considered as follows: (F) and (G) Dealloying reaction of Li*_x_*Sn (at ~0.45 V and ~0.65 V), (H) Dealloying reaction of Li*_x_*Sn and Li extraction to Li_3_P to form LiP (at ~0.75 V) and (I) Li extraction from Li*_x_*P (at >0.8 V). Labels (A)–(H) for these expected reactions are also plotted in a graph.

In the range of 20 cycles or less, the contributions of (A) and (B) in charge and (I) in discharge decrease remarkably with cycling, while the reactivity of Sn for (D) and Li*_x_*Sn for (G) seems to be activated with cycling. After discharging, Sn_4_P_3_ is not completely formed reversibly but amorphous Sn and P are formed [8]. Moreover, it is demonstrated that the reversibility of the P ↔ Li*_x_*P reaction is not good due to the large volume change and poor conductivity of the LiP phase [8,9,46,47], resulting in rapid capacity fading of the Sn_4_P_3_/C composite film during the initial 20 cycles. The d*Q*/d*V* profiles at the 20th and 100th cycle are similar but the peak intensity for (B), (C) and (D) in charge and (E), (G) and (H) in discharge decreases with the cycle progress. At the 150th cycle, these specific peaks in d*Q*/d*V* profile become smaller and broader compared to the profile at the 100th cycle.

**Figure 9:**
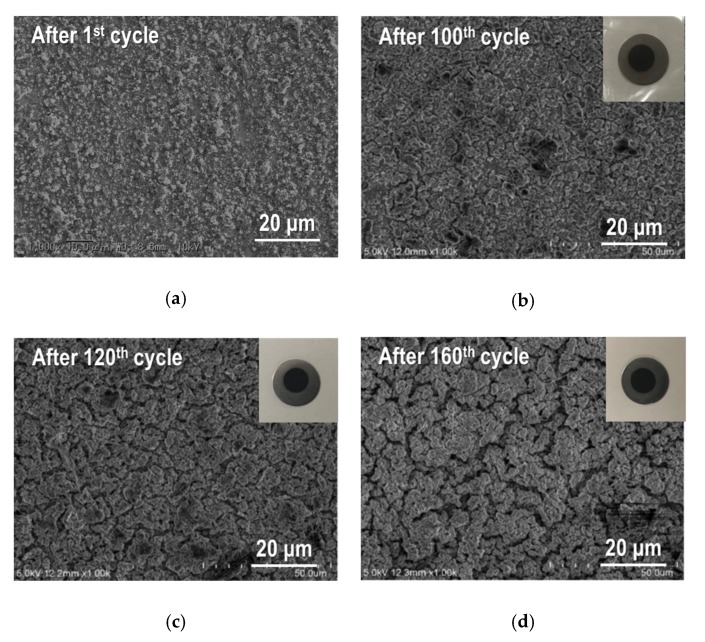
SEM images for the broader surface of Sn_4_P_3_/C composite films taken out of disassembled cells: (**a**) after the first cycle, (**b**) after the 100th cycle, (**c**) after the 120th cycle and (**d**) after the 160th cycle. Insets in (**b**), (**c**) and (**d**) are the photo images of films with different cycle numbers.

For further examination, we fabricated another Sn_4_P_3_/C composite film to observe the change of the microstructure during the galvanostatic cycling. After the galvanostatic testing with different cycles, we took out the film electrodes of the disassembled cells and confirmed that all the films were not delaminated from the SUS316L substrates (see insets in Figure 9). SEM images for Sn_4_P_3_/C composite film after the first, 100th, 120th and 160th cycle are summarized in Figure 9. It is worth noting that capacity fading behavior with cycling for all Sn_4_P_3_/C films is nearly the same as the data shown in Figure 7. After the first cycle (Figure 9a), it can be seen that the asperities on the surface of the composite film are clearly increased compared to the as-deposited film (Figure 5a). This is caused by the large volume expansion and contraction in the charge and discharge reactions of active material. Such structural change is repeated during cycling and induces the gradual capacity fading with cycling. At the 100th cycle (Figure 9b), generation of many small cracks and agglomeration of the particles are confirmed and become significant with further cycling (Figure 9c,d). The former breaks the electrical conduction path and the latter reduces to electrochemical utilization of active materials in the composite film, resulting in acceleration of the degradation of reversible capacities after 100 cycles as shown in Figure 7.

**Figure 10:**
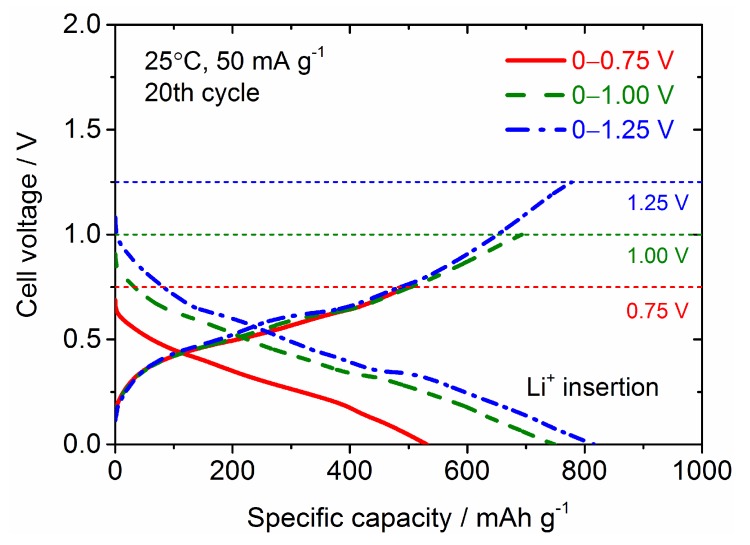
Comparison of galvanostatic charge (Li^+^ insertion) and discharge (Li^+^ extraction) curves at the 20th cycle for the Sn_4_P_3_/C composite film electrode tested at different cell voltage windows.

**Figure 11:**
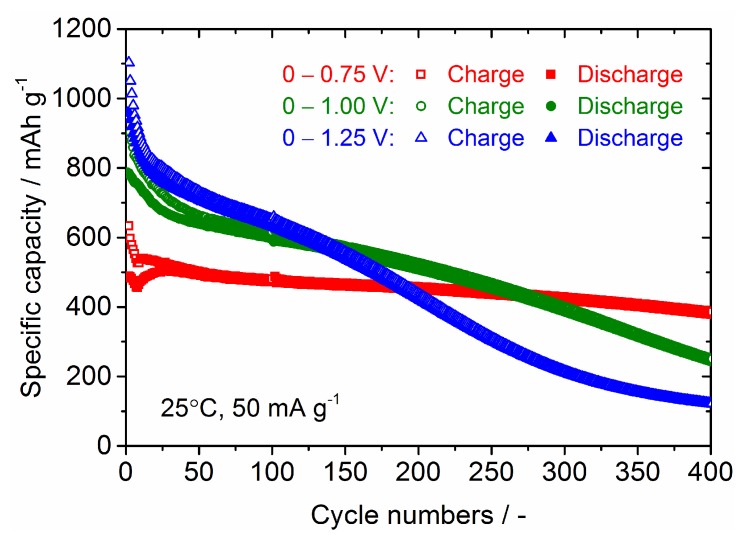
Cycling stability of charge and discharge capacities for the Sn_4_P_3_/C composite film tested at different cell voltage windows.

The improvement of the cycling stability of the Sn_4_P_3_ anode has been demonstrated by controlling the cell voltage window in the literature but the cycle numbers were limited to only 50 [8,9], so we investigated the long-term cycling stability for Sn_4_P_3_/C composite films at different cell potential windows of 0–0.75 V, 0–1 V and 0–1.25 V. The galvanostatic charge and discharge curves at the 20th cycle and cycling performance for Sn_4_P_3_/C composite films tested at different cell potential windows are shown in Figure 10 and Figure 11. Although the reversible capacities at the 20th cycle reduce monotonically with decreasing cell voltage for discharge from 1.25 to 0.75 V, the cycling stability is dramatically improved. The film tested at 0–0.75 V shows a reversible capacity of 380 mAh g^−1^ at the 400th cycle and the capacity retention reaches 80%. The film electrodes tested at 0–1 V and 0–1.25 V also show higher reversible capacities of 400 and 500 mAh g^−1^ at the 200th cycle than the film tested at 0–2.5 V (300 mAh g^−1^, see Figure 7), but the capacity fading is accelerated with further cycling. Reversible capacities at the 400th cycle for the films tested at 0–1 V and 0–1.25 V are only 250 and 110 mAh g^−1^, respectively. By limiting the potential for discharge below 0.75 V, the extraction reaction of Li from Li*_x_*P with a larger volume change is greatly suppressed, which could contribute to better cycling stability.

**Figure 12:**
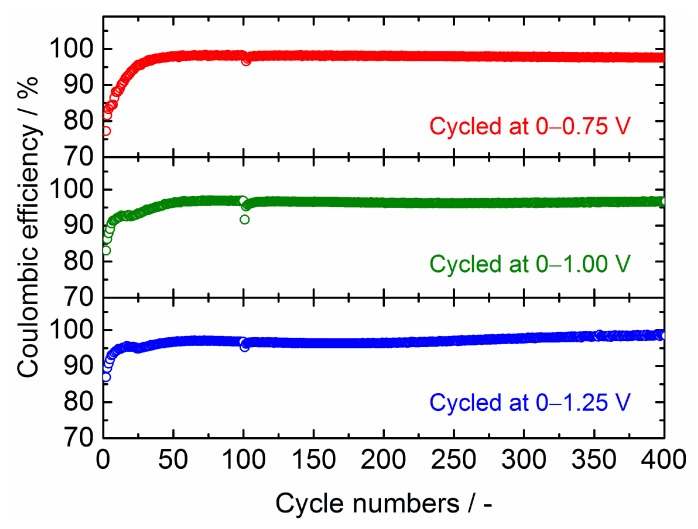
Coulombic efficiencies plotted against the cycle numbers for Sn_4_P_3_/C composite films tested at different cell voltage windows.

In Figure 12, the Coulombic efficiencies for Sn_4_P_3_/C composite films tested at different cell voltage windows are plotted against the cycle numbers. It can be seen that the efficiency for the initial several cycles becomes lower by reducing the cell voltage for discharging (Li extraction) from 1.25 to 0.75 V. This is mainly attributed to the reduction of the Li^+^ extraction reaction from Li*_x_*P that occurred at a cell voltage above 0.8 V. After 20 cycles, the efficiencies for all films reached approximately 95%. After 40 cycles, the film tested at 0−0.75 V shows an efficiency as high as 97−98% and maintains it stably in whole measurement range. This is consistent with the good cycling stability of this film (Figure 11). On the other hand, the films tested at 0–1 V and 0–1.25 V show a slightly lower efficiency of 96−97% after 40 cycles.

**Figure 13:**
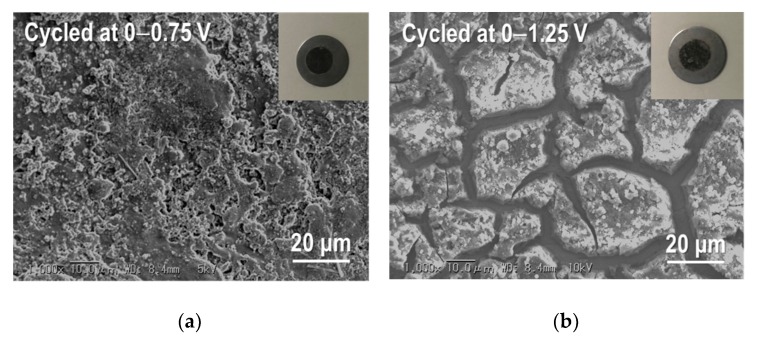
SEM images for the broader surface of Sn_4_P_3_/C composite films taken out of disassembled cells: (**a**) cycled at 0–0.75 V and (**b**) cycled at 0–1.25 V. Insets are the photo images of films.

For further examination, we took the Sn_4_P_3_/C composite film electrodes out of the disassembled cells after cycling at different cell voltage windows and observed their microstructures by SEM (Figure 13). As shown in Figure 13a, no delamination of the film from the SUS316L substrate was observed after cycling at 0–0.75 V. In addition, the structural change of the film cycled at 0–0.75 V is less than that for the film cycled at 0–1.25 V, which contributes to the better cycling performance. For the film cycled at 0–1.25 V with the lowest capacity retention (Figure 13b), delamination of many parts of the film from the substrate is confirmed and the large transversal cracks were generated at the location without peeling.

Lastly, we compare the electrochemical performance of some Sn_4_P_3_ anode materials for LiBs reported in the literature [8,9,11,14,15,16,17,20,21,22,23,24], which is listed in Table 1. It is worth noting that our current results reported in this paper are not the top performance for Sn_4_P_3_ anode materials for LiBs. As reported in [21], Sn_4_P_3_/C nanospheres synthesized by carbonization/reduction and phosphorization of SnO_2_–GCP (glucose-derived, carbon-rich polysaccharide) nanospheres showed an outstanding rate performance and cycling stability. These nanospheres can also be applicable for ultra-stable anode materials for sodium-ion batteries (SiBs). However, as mentioned above, the electrodes with these Sn_4_P_3_/C composite anode materials used in batteries are fabricated by a slurry coating process with a large amount of carbon additives and binders [20,21,22,23,24], resulting in the decrease of the fraction of Sn_4_P_3_ in the electrode. For the Sn_4_P_3_/C composite anode listed in Table 1, gravimetric capacities calculated by the total mass of electrode (including both carbon additives and binders) are 20−30% lower than the listed values. On the other hand, Sn_4_P_3_/C composite film electrodes formed by AD do not contain other carbon additives and binders, resulting in higher gravimetric capacity for the whole electrode. There is room to improve the electrochemical performance of the size and content of carbon materials for Sn_4_P_3_/C powders used for AD. We are now trying to optimize complexed carbon content and increase the composite film thickness and the progress will be reported in a forthcoming paper.

## 4. Conclusions

Sn_4_P_3_/C composite film was successfully fabricated by the AD method and its electrochemical performance for a lithium-ion battery anode was examined. The Sn_4_P_3_/C composite film fabricated by AD showed much better electrochemical performance than the Sn_4_P_3_ film without complexing carbon. Although both films showed initial discharge (Li^+^ extraction) capacities of approximately 900–1000 mAh g^−1^, Sn_4_P_3_/C films retained the higher reversible capacity above 700 mAh g^−1^ after 100 cycles of charge and discharge processes while the capacity of the Sn_4_P_3_ film rapidly degraded with cycling. Precise control of the potential window in galvanostatic testing of the Sn_4_P_3_/C composite film results in remarkable improvement in the cycling performance. We obtained a reversible capacity of approximately 400 mAh g^−1^ after 400 cycles by controlling the cell potential window, which is mainly attributed to the suppression of structural change of the film electrode during the cycling.

## Figures and Tables

**Table 1:** Comparison of electrochemical performance of some Sn_4_P_3_ anode materials for Li-ion batteries (LiB). AD: aerosol deposition.

Samples	Current Density/mA g^−1^	Cycle Numbers	Specific Capacity/mAh g^−^^1^	References
Sn_4_P_3_	100	50	370	[8]
Sn_4+x_P_3_	100	50	530 (*x* = 1) 430 (*x* = 0.5)	[9]
Sn_4_P_3_ film by pulsed laser deposition (PLD)	0.2 mA cm^−2^	10	553	[11]
Fe doped Sn_4_P_3_	100	100	420	[14]
Mn doped Sn_4_P_3_	100	150	488	[15]
Sn_4_P_3_	100	20	261	[16]
Sn_4_P_3_	100	300	442	[17]
Sn_4_P_3_/graphite	100	100	651	[20]
Sn_4_P_3_/C nanosphere	200 2000	50 500	1050 440	[21]
Sn_4_P_3_/SnO_2_–C	400	200	733	[22]
Sn_4_P_3_/hollow graphene sphere	100	100	606	[23]
Sn_4_P_3_/N doped C	100	120	718	[24]
Sn_4_P_3_/C film by AD	50	100 400	726 380	This work

## Supplementary Materials

The following are available online at https://www.mdpi.com/2079-4991/9/7/1032/s1, Figure S1: (**a**) SEM image of broader surface (left) and transverse cross section (right) of the Sn_4_P_3_ film fabricated by AD and (**b**) Galvanostatic charge and discharge curves for the Sn_4_P_3_ film, Figure S2: (**a**) Galvanostatic charge and discharge curves for the Sn_4_P_3_/C composite film (Sn_4_P_3_:AB = 9:1 in weight) and (**b**) Comparison of cycling stability for Sn_4_P_3_/C composite films with different carbon content.
