# Supplementary material for: Characterization of Sn4P3–Carbon Composite Films for Lithium-Ion Battery Anode Fabricated by Aerosol Deposition
Source: Nanomaterials (Basel). 2019 Jul 19;9(7):1032. doi: 10.3390/nano9071032 (PMC6669548; doi:10.3390/nano9071032)
Supplement: Supplementary file 1 [file nanomaterials-09-01032-s001.pdf]

Supplementary Information

Characterization of Sn_4P_3–Carbon Composite Films for Lithium-Ion Battery Anode Fabricated by Aerosol Deposition

Toki Moritaka [1], Yuh Yamashita [1], Tomohiro Tojo [2], Ryoji Inada [1,*] and Yoji Sakurai [1]

[1] Department of Electrical and Electronic Information Engineering, Toyohashi University of Technology, 1-1 Tempaku-cho, Toyohashi, Aichi 4418580, Japan

[2] Department of Electrical and Electronic Engineering, Shizuoka Institute of Science and Technology, 2200-2 Toyosawa, Fukuroi, Shizuoka 437-8555, Japan

* Correspondence: inada@ee.tut.ac.jp; Tel.: +81-532-446723

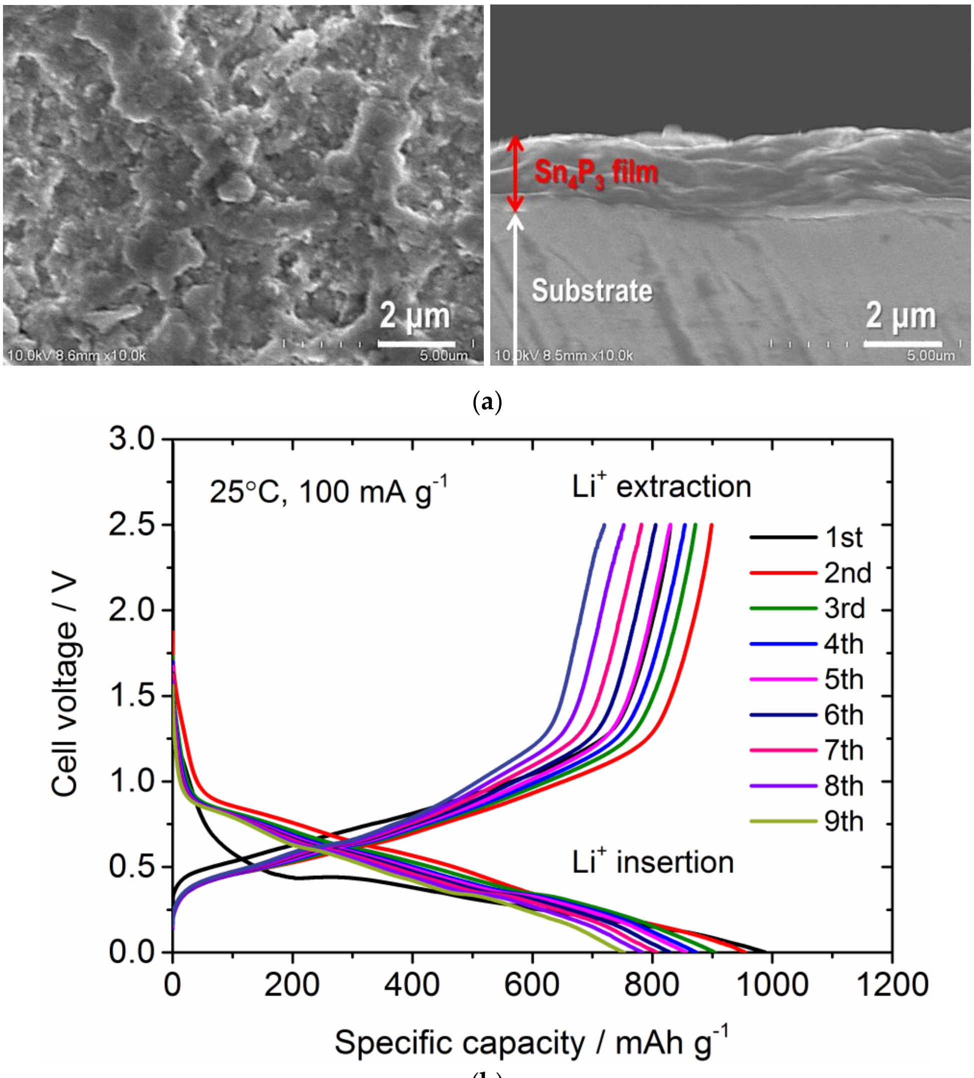

(**b**)

Figure S1. (**a**) SEM image of broader surface (left) and transverse cross section (right) of the Sn_4P_3 film fabricated by AD and (**b**) Galvanostatic charge and discharge curves for the Sn_4P_3 film.

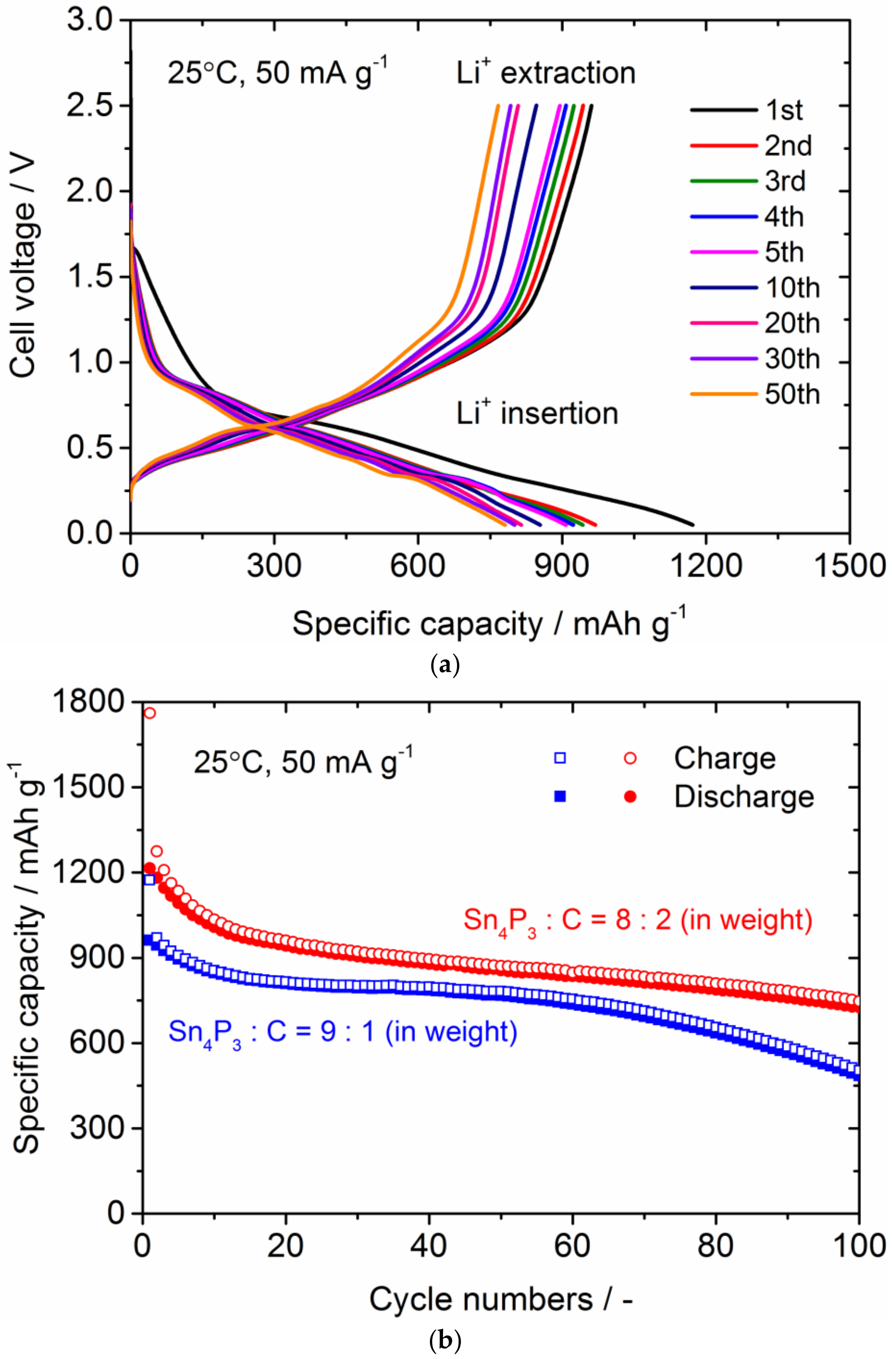

Figure S2. (**a**) Galvanostatic charge and discharge curves for the Sn_4P_3/C composite film (Sn_4P_3:AB = 9:1 in weight) and (**b**) Comparison of cycling stability for Sn_4P_3/C composite films with different carbon content.
